# A study of stress response to endotracheal intubation comparing glidescope and flexible fiberoptic bronchoscope

**DOI:** 10.12669/pjms.305.4788

**Published:** 2014

**Authors:** Mansoor Aqil

**Affiliations:** 1 Mansoor Aqil, FCPS, Associate Professor, Department of Anesthesiology (41), College of Medicine, King Saud University, Consultant Anesthetist, King Khalid University Hospital, Riyadh, Saudi Arabia.

**Keywords:** Glidescope, Flexible fiberoptic laryngoscope, Intubation response, Endotracheal intubation, Hemodynamic stress response

## Abstract

***Objective: ***To compare hemodynamic stress response (HDSR) to ET intubation using Glidescope (GLS) and Flexible fiberoptic laryngoscope (FFB).

***Methods: ***This prospective randomized comparative study was conducted at King Khalid University Hospital, King Saud University, Riyadh, Saudi Arabia from June 2011 - November 2013. Eighty ASA 1 & 2 patients with normal airway undergoing elective surgical procedure requiring ET intubation were included in the study. Patients were randomly assigned in two groups GLS or FFB. General anesthesia was induced with propofol and fentanyl. Muscle relaxation was achieved with cisatracurium and ET intubation was performed using either GLS or FFB. Noninvasive hemodynamic data was recorded (HR, systolic, diastolic and mean blood pressure) as pre-induction, baseline and after ET intubation at one minute intervals for successive five minutes. End tidal Sevoflurane and CO2 at the time of intubation, need of external neck pressure, time to successful intubation and number of attempts were recorded; and rate pressure product was calculated.

***Results: ***Induction of anesthesia resulted in significant fall in blood pressure in both the groups. ET intubation resulted in similar rise of BP in both groups (for 3-4 minutes) from their baseline values; however the rise was not significantly different from their respective pre-induction values. Time to intubation was longer with FFB compared to GLS however, need for external neck manipulation was more with GLS.

***Conclusion: ***There was no difference in HDSR due to ET intubation using either GLS or FFB in healthy adult patients with normal airway. Rate pressure product remained within the acceptable range.

## INTRODUCTION

Manipulation of the airway during endotracheal (ET) intubation leads to stimulation of pharyngeal and tracheolaryngeal nociceptors resulting in hemodynamic stress response (HDSR),^1^ which can be deleterious in patients with poor cardiac reserves^2^ or having other comorbidities.^3^ The magnitude of the HDSR is variable and proportional to the amount of force applied during visualization of the glottis^4^ and the degree of tracheolaryngeal manipulation during advancement of ET into the trachea.^5^

There is some equipment available for ET intubation in which indirect laryngoscopy is used and application of upward and forward force is not required during visualization of the glottis and requires variable degree of airway manipulation during advancement of the ET tube.^6^^-^^9^ Glidescope (GLS) and flexible fiberoptic bronchoscope (FFB) are two novel instruments, requiring minimum to no force during visualization of the glottis and provide an improved view.^10^

However, literature shows conflicting results regarding the magnitude of said HDSR induced during ET intubation using GLS in comparison with FFB.^11^^-^^13^ The objective of our prospective randomized study was to compare the HDSR during ET intubation using GLS and FFB in our setting.

## METHODS

Approval of the Hospital committee of Ethics on Research was taken. Eighty adult patients ASA physical status 1&2 and BMI between 25-30, planned to undergo elective surgery, requiring general anesthesia and ET intubation were included in the study and signed informed consent. Their ages were between 18-60 years and Mallampati class was 1&2. Patients with anticipated/history of previous difficult intubation or mask ventilation, cervical spine injury, raised ICP, history of gastro-esophageal reflux requiring rapid sequence induction, or those on any regular drug intake or allergy to any anesthetic drug used in the study were excluded. 

In all the patients, preoperative fasting was for 6-8 hours and each received oral premedication lorazepam in the dose of 2mg (tablet) about 2 hours before surgery. At the same time an intravenous (i.v) line was established and Ringer’s lactate infusion at the rate of 15 ml.kg-1.hr-1 was started through an infusion pump. In the operating room, all the patients were lying supine with head placed on a regular pillow (4-6 inches height). Monitoring was standard noninvasive (ECG, HR, systolic (S) blood pressure (BP), diastolic (D) BP, mean (M) BP, oxygen saturation by pulse oximetry (SPO2), end tidal carbon dioxide (CO2) and end tidal sevoflurane (SEV). Hemodynamic recording was done as pre-induction, prior to intubation of anesthesia (baseline) and subsequently after every minute post intubation for 5 minutes. All the patients were preoxygenated for 3 minutes with 100% oxygen through gently placed anesthesia face mask and received i.v glycopyrolate in the dose of 0.2 mg; followed by standardized induction of anesthesia with i.v fentanyl 2 microgram.kg-1 and propofol 2 mg.kg-1. Ability to ventilate the lungs was assessed using manual ventilation with face mask and after confirmation; muscle relaxation was achieved with cisatracurium in the dose of 0.15mg.kg-1. Till the establishment of complete neuromuscular block (assessed by absence response to train of four stimulation of ulnar nerve), ventilation of the patient was gently assisted manually with intermittent positive pressure ventilation (IPPV) using a mixture of 2% SEV in 100% oxygen. End tidal CO2 was maintained between (30-35 mm Hg) during IPPV; and recorded immediately after intubation and then continuously afterwards. All the intubations were performed by a well-trained anesthetist with >20 years’ experience in the specialty and having > 100 successful intubations with both the instruments. The attempt was stopped if it exceeded 120 seconds, SPO2 dropped below 92%, or resulted in airway trauma (i.e., blood stain on the laryngoscope blade).Manual IPPV with the same anesthetic mixture was done between the attempts. It was considered a failed intubation if two attempts were unsuccessful in ET intubation or in case of equipment malfunction after which failed intubation algorithm was followed.^14^

Anesthesia delivery unit used in the study was Datex Ohmeda Aisys® workstation (DOWS). End tidal SEV was measured during manual IPPV, at the time of intubation and immediately post-intubation; and then it was maintained at 2% through end tidal control mode of the DOWS. The time to intubation (TTI) was calculated from the time the instrument entered the patient’s oral cavity until end-tidal CO2 tracing was detected on the monitor after commencement of IPPV. Rate pressure product (RPP) was calculated as the product of HR and SBP at the above mentioned timings.

Randomization of patients in the two groups was done using an online software (http://www.randomizer.org/form.htm). Laryngoscopy and ET intubation was done according to the patient’s group. For ET intubation with GLS, size 4 blade was used in all of the cases. GLS was advanced gently in the oral cavity (in the midline) and walked down the tongue. The scope was further advanced into the vallecula and gentle lifting force was applied for visualization of the glottis. ET tube was pre-loaded on the specific rigid stylet (GlideRite having 60 degree angulation) and was advanced into the trachea by the primary operator. External pressure to the front of the neck was applied on request of the operator by another anesthetist. Size 6.0 OD bronchoscope (Olympus) was used in FFB group. Intubation with FFB was done through an orally inserted William’s intubator oral airway® size 9 in female and size 10 in male patients. After development of adequate muscle relaxation, the airway was inserted into the oral cavity and positioned in the midline while one anesthetist performed the jaw thrust maneuver. FFB preloaded with proper size ET tube was inserted (by the primary operator) through the airway into the glottis and trachea. The FFB was advanced till bifurcation of the trachea was visualized then the ET tube was railroaded over the FFB into the trachea and the scope removed gently while viewing the tracheal rings and ET tube.

In both the groups, size 7.0 mm internal diameter (ID) ET tube which was well lubricated with water soluble KY Jelly (Johnsons and Johnsons®) was used in females and 8.0 ID in male patients. After successful intubation, the cuff of the ET tube was inflated with air to a pressure of 20 mm Hg and proper placement of ET tube was confirmed by capnography tracing. Afterwards, anesthesia was maintained with 2% SEV (through end tidal control of DOWS) in 50% oxygen air mixture. The ventilatory parameters were, tidal volume 8-10 ml.kg-1 and respiratory rate (8-12 min-1) adjusted to keep end tidal CO2 between 30-35 mm Hg. No other medication was administered for the next five minutes during the period of data collection and the patients were not stimulated at all. Number of attempts and lowest SPO2 during the process of ET intubation was recorded. After five minutes of data collection, subsequent anesthesia management was according to the surgical plan and on the discretion of the anesthetist.

***Statistical analysis:*** Our data was analyzed by SPSS statistical software (SPSS Inc, Chicago, IL) version 17.0. Descriptive data were analyzed by two-tailed Student’s *t*-test. HR and BP analysis was performed using two-tailed independent *t*-test. Chi-square test or Fisher’s exact test was used to analyze categorical variables. Bonferroni-Dunn test was used to compare mean values between the groups. The quantitative data was expressed as mean ± standard deviation (SD). A value of P < 0.05 was considered statistically significant.

Sample size and power of analysis was calculated on the basis of our pilot study to detect 20% difference in BP and HR for type I error of 0.05 and type II error of 0.2 and power of 0.8, hence we chose 40 patients in each group.

## RESULTS

There was no significant difference between the groups in age, sex, weight, height, BMI, pre-induction HR, SBP, DBP, MAP, number of attempts for successful intubation, lowest SPO2 during intubation, end tidal CO2 at the time of intubation, end tidal SEV at the time of intubation and other airway parameters(Table-I). Time to successful intubation was more in FFB group while need for external manipulation of neck was more in GLS group (Table-I).

Induction of anesthesia resulted in significant fall in SDP, DBP and MAP in both the groups from their pre-induction values; however, the drop in said parameters was similar in both groups (Fig. 2, 3 and 4).

**Fig.1:**
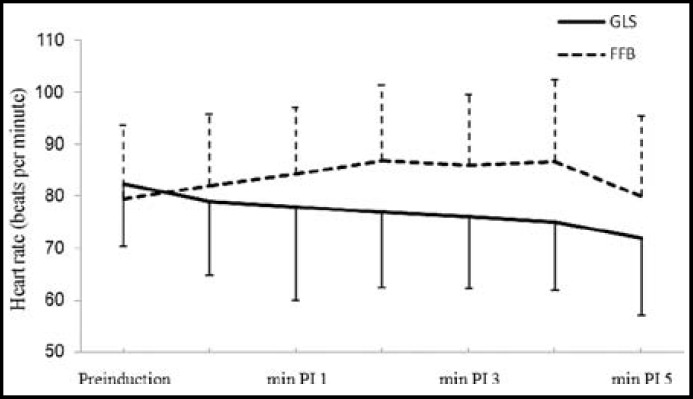
Changes in heart rate associated with endotracheal intubation

**Fig.2:**
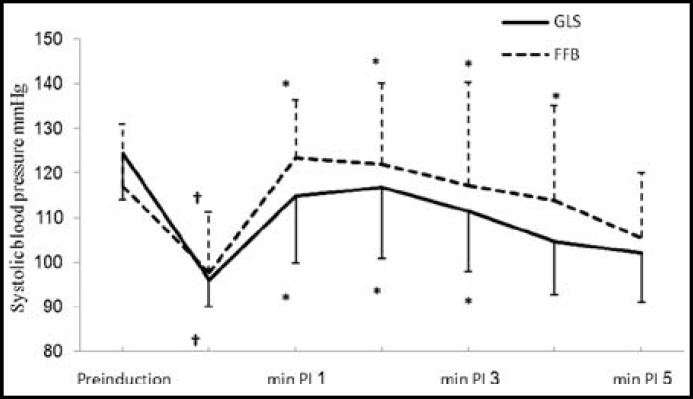
Changes in systolic blood pressure associated with endotracheal intubation

**Fig.3:**
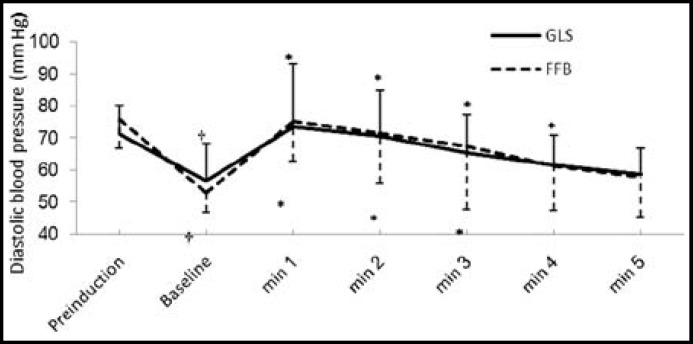
Changes in diastolic blood pressure associated with endotracheal intubation

**Fig.4:**
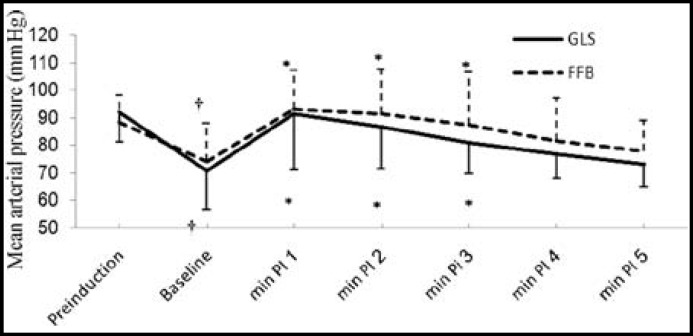
Changes in mean arterial pressure associated with endotracheal intubation

**Fig.5:**
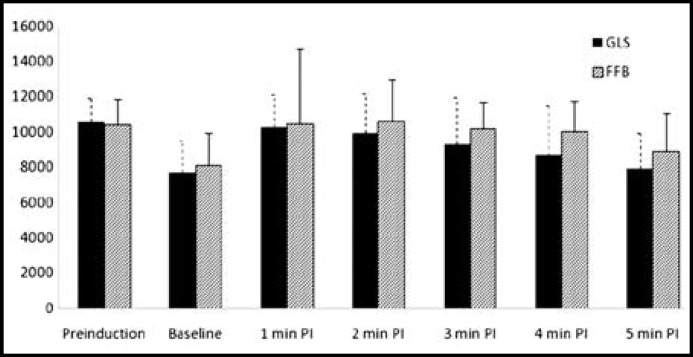
Changes in rate pressure product associated with endotracheal intubation

There was significant rise in SBP from the baseline values for 3 minutes in GLS group and for 4 minutes in FFB; however, it was not significantly different from their pre-induction values (Fig.2). There was significant rise in DBP from respective baseline values which lasted for four minutes in FFB and for three minutes in GLS group, however, there was no substantial difference from their respective baseline values (Fig.3). ET intubation resulted in comparable rise in MAP in both the groups which lasted for 3 minutes (Fig.4). None of the patients in any group developed severe hypotension (SBP <90 mmHg) or severe hypertension (SBP > 170 mmHg). There was no significant change in HR and RPP from the pre-induction values in both the groups throughout the study period (Fig. 1 and 5).

## DISCUSSION

In this study, we did not find any significant difference in HDSR using either, GLS or FFB. Similar to our results Xue FS and colleagues did not find any difference in HDSR with GLS and FFB.^11^ However, contrarily, Nataļja and coworkers^12^ found significantly increased HDSR with FFB in comparison to GLS. There are two main components that determine HDSR, these are, the degree and the duration of pharyngeal and tracheolaryngeal stimulation.^5^^,^^15^ In our view, the cause of higher HDSR response with FFB in their^12^ study was prolonged TTI, which was more than double compared to that of our patients (120 v.s 59 seconds) and resulted in extended stimulation of the airway. The reason for prolonged TTI in their study was due to their encountering difficulties in visualizing the glottis leading to greater airway manipulation. The authors have related the difficulty to the use of muscle relaxants which resulted in slackening of the tongue, pharyngeal muscles and epiglottis. Although in our patients, we also used muscle relaxants, we did not encounter the same difficulty. The reason for the difference in results is certain dissimilarities in the design and technique of ET intubation in both the series. Firstly, we used William’s oral airway in our patients; this airway provided an unobstructed channel for entry of the FFB, guided it towards glottis, improved visualization^16^, and decreased TTI and the duration of airway manipulation. Secondly, one anesthetist applied the jaw thrust maneuver to all our patients in FFB group. This maneuver has been proven to make FFB intubation easier.^17^

**Table-I:** Demographics, baseline hemodynamic data and results

	***Patient group*** ***N= 40***	***Mean ± SD***	***P Value***
Age (years)	GLS	38.03 ± 12.03	0.4
	FFB	40.45 ± 10.33	
Weight (Kg)	GLS	74.8 ± 12.44	0.89
	FFB	75.2 5± 13.77	
Height (cm)	GLS	162.54 ± 7.5	0.53
	FFB	161.16 ± 9.58	
Sex (F/M)	GLS	17/23	
	FFB	18/22	
Body mass index (BMI )	GLS	28.3 ± 1.7	0.87
	FFB	28.93 ± 1.1	
◊Heart rate (HR) b.p.m	GLS	85.15 ± 12.08	0.21
	FFB	89.26 ± 11.69	
◊Systolic Blood pressure mm Hg	GLS	119.26 ± 9.32	0.255
	FFB	115.15 ±15.46	
◊Diastolic Blood pressure mm Hg	GLS	77.19 ± 10.63	0.64
	FFB	75.92 ± 9.05	
◊Mean Blood pressure mm Hg	GLS	91.07 ± 8.47	0.29
	FFB	88.34 ± 10.03	
Mallampati class 1/2/3/4	GLS	33/6/1/0	
	FFB	31/7/2/0	
Thyromental distance (mm)	GLS	77.4 ± 9.5	0.82
	FFB	78. 6± 8.9	
Mouth opening (mm)	GLS	38.7 ± 5.3	0.64
	FFB	40.2 ± 3.6	
Time to intubate (sec)	GLS	43.55 ± 22.59	0.04*
	FFB	58.96 ± 36.84	
Number of attempts for intubation	GLS	1	
	FFB	1	
Lowest SPO2 (%) at the time of ET intubation	GLS	95.2 ± 3.2	0.75
	FFB	94.7 ± 2.6	
End tidal CO2 at time of intubation (mm Hg)	GLS	35.8 ± 2.1	0.63
	FFB	37.6 ± 1.6	
End Tidal SEV at the time of intubation (%)	GLS	1.7 ± 0.24	0.81
	FFB	1.6 ± 0.31	
Need of external neck manipulation	GLS	9	0.0005*
	FFB	1	

GLS= Glidescope, FFB= flexible fiberoptic bronchoscope, N= number of patients in each group,

SD= Standard deviation. All values expressed as mean ± Standard deviation, CO2 = carbon dioxide, SEV= sevoflurane, b.p.m = beats per minute, SPO2 =Oxygen saturation by pulse oximeter,

* = P value <0.05

◊ = Pre-induction

GLS= glidescope, FFB= flexible fiberoptic bronchoscope, PI = post intubation, mean values are shown with standard deviation (SD).

Although our TTI with FFB was less compared to other researchers^12^, it was more compared to the GLS group in this study. The reason for greater TTI with FFB guided ET placement includes various intraoral and intratracheal maneuvers like bending its tip to visualize the glottis, gentle advancement and entry into the trachea, insertion of FFB usually deep into the trachea, rotation of ET tube while advancement (as it is usually stuck at the level of cords) and finally, removal of the FOB. Some studies have demonstrated that the tracheal stimulus is an important contributor leading to HDSR during FFB intubation.^13^^,^^18^ In our study, FFB was also inserted till the bifurcation of trachea was seen which, consequentially, required all above mentioned maneuvers, thus leading to increased TTI. Moreover, we also applied the jaw thrust maneuver that leads to HDSR.^19^ Despite the greater TTI leading to increased tracheal stimulation and application of jaw thrust maneuver in FFB group, HDSR was still not higher than the GLS group. This can be explained by the contribution of the need for external neck manipulation (despite getting excellent view of the glottis) in GLS group leading to a high HDSR. Moreover, we encountered difficulty in advancement of the ET tube through the glottis due to the curvature of the blade and the stylet as the ET tube snagged on the anterior wall of the trachea, requiring more manipulation and external pressure on the neck in the backward, upward and right direction. Other investigators also encountered the same difficulty while using GLS.^8^^,^^20^^,^^21^ In our opinion, the application of force on GLS handle in the forward direction (approximately 7.6 Newton) during visualization of glottis^22^ and the need of external neck manipulation resulted in airway stimulation and contributed to HDSR and thus the net result was comparable HDSR in both the groups.

Laryngoscopy and ET intubation affects the HR, causing tachycardia^1^, contrarily we did not find any effect of ET intubation on HR in either group. In our opinion, it was due to the combined effect of fentanyl, propofol and SEV on the HR.^23^

After induction of anesthesia there was a significant and equivalent fall in SBP, DBP and MAP from their respective pre-induction values in both groups. The fall in BP was due to combined effect of fentanyl, SEV, propofol and hypovolemia secondary to fasting and institution of IPPV.^24^

RPP is an index of myocardial oxygen consumption and a value above 22000 is considered to be detrimental to the heart.^25^ Our results show that although SBP increased about 27% in FFB and 21% in GLS group, the maximal RPP value in either group did not exceed 15000 and was maintained within acceptable limits.

*Limitations of the study:* All the ET intubations were performed by one anesthetist and we acknowledge that the potential of bias exists, however; due to the design of the study it was not possible to blind the operator or observer. To overcome this issue, we selected a reasonably well experienced operator and most of the data was recorded and printed by an electronic device, potential of bias does not exist. Secondly, our results may differ from that of another anesthetist having a different level of skill and experience. Thirdly, all the patients with predicted difficult intubation were excluded from the study and consequently our data may not be applicable to patients with anticipated or actual difficult intubation. Fourthly, all the patients in this study were ASA physical status 1&2 and our results do not reflect the HD changes in patients with comorbidities. Furthermore, our patients were hypovolemic because of a fasting period of about 4 hours and received maintenance fluids only 2 hours prior to data collection. The response might have been different if these patients were euvolemic; however, hypovolemia affected patients in both the groups in a similar manner. Finally, we used noninvasive BP recording whereas invasive BP recording would have been superior; nevertheless, it was ethically unacceptable to have used an invasive BP recording for study purposes only.

## CONCLUSION

Our results reveal that ET intubation using FFB or GLS does not lead to any significant HDSR that can be detrimental in patients with normal airway. Further studies are needed to find out the HDSR in patients with difficult intubation and with comorbidities. 

## Notes

***Grant Support & Financial Disclosures:*** None (This study was conducted without any funding or financial assistance from any source).
